# Progress in Anæsthetics

**Published:** 1902-07-19

**Authors:** 


### Progress in Anæsthetics.

Accidents occurring after anæsthetics have been used, although not frequent relatively to the number of times anæsthetics are administered, yet are too frequent to allow of its being said that they are unavoidable. With reference particularly to the occurrence of lung complications after anæsthesia, Crouch and Corner[1] have examined all the cases coming under anæsthesia for twelve months at St. Thomas's Hospital. These comprised 3,000 cases, being 2,400 by ether and 600 by chloroform. None of the latter but 10 of the former showed any lung complications, and one of these died. They were healthy persons, with no bad lung history, they gave no trouble to the anæsthetist nor became cyanosed, blocked with mucous or the like. They were long operations, mostly trunk cases, though one was varicose veins; 7 of the 10 were in summer; 7 suffered from bronchitis and 3 of broncho-pneumonia. The authors put the accidents down to the ether primarily, secondarily to exposure and tight bandaging. Possibly the previous use of too much nitrous-oxide gas helped to a bad effect. Dudley Buxton considers tight bandaging to be deplored after ether, but that the troubles arose from ether being continued too strong after anæsthesia had been reached.

Anything which will lessen vomiting after ether will add to its safety. Numerous remedies and methods have been advocated from time to time without success being constant. The opinion is gaining ground that nausea and vomiting are caused by the irritation of the gastric mucous membrane not by the ether swallowed, so much as by that excreted into the stomach by the mucous membrane itself, and that it is a condition of gastritis. A rational method therefore of prevention would be one which would aim at so diluting the ether in its transit that it should become non-irritant. This is the method recommended by Hess[2] who argues that the usual method at present adopted in the preparation of the patient for ether anæsthesia is to restrict fluids very much with the food and to deplete the system of water by means of salines, enemas, and aperients generally. The patient comes to the table with parched lips and with urine of a high specific gravity; and hence not in a good condition to excrete any large amount of irritant poison. A good deal can be done by lessening the amount of ether taken by the use of a closed inhaler, and especially by never allowing the vapour to be inhaled in too concentrated a form; and nitrous oxide gas is an excellent adjuvant in producing the anæsthesia; but the method he would urge is the free use of water before and after the anæsthetic. In this way vomiting has effectually been prevented in not a few cases. He uses 6–10 oz. before and plenty to drink as soon as consciousness returns. But there are other evils than vomiting attendant upon the administration of ether —the drug itself has apparently an effect upon the blood-cells, for, according to Da Costa and Kalteyer,[3] polycythæmia (rarely oligocythæmia) has been noticed in fifty cases examined by them. This may be partly due to the inspissation of the blood from preanæsthetic preparation and sweating, but the phenomena disappeared on discontinuing the anæsthetic. The hæmoglobin is reduced actually in amount in the cells, and the authors consider that there is an increase of hæmolysis. The effects were not due to hæmorrhage, as in only a few cases was there a considerable loss of blood. They suggest that the blood of patients should be examined before all operations. Should the hæmoglobin be less than 50 per cent. there is great risk in giving an anæsthetic. The saturation of the tissues with an anæsthetic has been found to produce apparent degeneration of the muscular tissue and visceral degeneration (Strasbourg, Fischer and Thiem), but Hamilton Wright[4] has found pseudo-degenerative changes in the neurons of animals under chloroform, and to a less extent under ether. The changes that are marked in a rabbit are shown to a less degree in a dog, and the presumption is that in man there are still less again. These profound changes are not dependent upon congestion, nor are they permanent, for after six hours' prolonged anæsthesia they do not last forty-eight hours.

Maurice Arthus[5] considers that the irritant action of chloroform upon the mucous membranes is due to impurities or to a too concentrated vapour suddenly inhaled when there is a state of exaggerated reflex excitability. As Brodie and Russell[6] find with electrical stimulation of the nasal and laryngeal mucous membranes under A.C.E. or urethane, so

Arthus finds with concentrated chloroform vapour that the heart is markedly slowed, leading to cardiac, and when the respiration is affected, respiratory syncope. This he terms laryngo-reflex or primary syncope. Both observers agree that the tracheal mucous membrane is not so sensitive, and according to Arthus this condition is never produced in a rabbit if the chloroform vapour is administered through a tracheal cannula. Electrical stimulation of the central ends of the vagus branches, and especially the pulmonary branches, and also the alveolar nerves under anæsthetics produces similar cardiac arrest (Brodie and Russell). If the anæsthetic be pressed too rapidly or suddenly, then there first occurs cardiac acceleration, then slowing, and lastly, secondary or "bulbar cardiac" syncope (Arthus). If the vagi of a dog be first cut, or atropine used, then the final syncope does not occur. If chloroform be pressed, paresis and then paralysis of the respiratory centre appear, "respiratory syncope" or "toxic apnœa," and breathing ceases before the heart stops beating.

To avoid these results in the use of ether or chloroform, Arthus recommends that we should administer the anæsthetics gradually, and lessen reflex excitability by means of morphia or chloral, and give atropine, which, by suppressing the cardiac moderator apparatus, suppresses primary syncope. Sudden increments should never be allowed, and the quantity should be lessened on the disappearance of the oculopalpebral reflex, and stopped if the pulse gets slow or the respiration weak. Primary and secondary cardiac syncope are irremediable, resulting from excitation of the moderator apparatus, but they can be prevented by atropine. He deplores the use of large and unmeasured doses, but advocates the method by which the dosage is regulated by means of drops: one, two, or three drops being allowed per breath during the process of "anæsthetism," while two or three drops per minute are sufficient for maintenance. Morphia economises the anæsthetic, depresses the cerebral centres, and stops the liability to primary syncope. The drug is accused of respiratory syncope; this is doubtful, but if so, it is the smaller danger of the two, for it can be obviated by artificial respiration. Chloral is not a good substitute for morphia as it predisposes to the irremediable cardiac syncope. He recommends most strongly the atropomorphine-chloroform method, with which accidents never happen in dogs. He gives one centigramme of morphine and one milligramme of atropine per kilogramme of body weight. In man 20–30 minutes prior to administration $1\frac{1}{2}$ c.c. of solution containing 10 centigrammes of morphine and 5 milligrammes of atropine sulphate are injected. This has been used hundreds of times safely, the post-operative sleep induced being rather beneficial than otherwise.

MacWilliams[7] has studied the effect of light or deep narcosis on the mammalian heart. At first the vagus centre is depressed with the corresponding acceleration of the pulse, then follows a slowing, with increased activity of the vagus. At a certain stage, he says, in chloroform anæsthesia the vagus centre becomes very sensitive and is prone to become affected by peripheral stimuli. Sudden arrest of respiratory movements leads to slowing and irregularity of pulse. In light narcosis, slowing, often slight, is followed by acceleration and marked convulsions. In deeper narcosis acceleration occurs with respiratory effort and motor excitement. In profound narcosis little change occurs at first, then retardation, but no convulsion.

Not a few have now testified to the value of ethyl chloride as a safe general anæsthetic. To these Marshall[8] adds his experience obtained in 46 dental cases, with two failures. He has had no cases of anxiety, and considers the anæsthetic an ideal one in children. Six of his cases were under ten years of age. He warns surgeons against being alone with female patients during its administration, as twice he has been accused of misconduct.

[1] Brit. Med. Jour., Jan. 18, 1902. [2] Med. Rec., Feb. 22. [3] Lancet, Dec. 28, 1901. [4] Lancet, Dec. 28, 1901. [5] Treatment, February 1902. [6] Lancet, Dec. 28, 1901. [7] Lancet, Dec. 28, 1901. [8] Treatment, February 1902.

